# Effects of Cigarette Smoke Condensate on Growth and Biofilm Formation by *Mycobacterium tuberculosis*

**DOI:** 10.1155/2020/8237402

**Published:** 2020-08-18

**Authors:** Moloko C. Cholo, Sipho S. M. Rasehlo, Eudri Venter, Chantelle Venter, Ronald Anderson

**Affiliations:** ^1^Department of Immunology, Faculty of Health Sciences, University of Pretoria, Pretoria, South Africa; ^2^Department of Medical Microbiology, Faculty of Health Sciences, University of Pretoria, Pretoria, South Africa; ^3^Laboratory for Microscopy and Microanalysis, Faculty of Natural and Agricultural Sciences, University of Pretoria, South Africa; ^4^Institute for Cellular and Molecular Medicine, Department of Immunology, Faculty of Health Sciences, University of Pretoria, Pretoria, South Africa

## Abstract

**Materials and Methods:**

The planktonic and biofilm-forming cultures were prepared in Middlebrook 7H9 and Sauton broth media, respectively, using *Mtb* strain, H37Rv. The effects of CSC at concentrations of 0.05-3.12 mg/L on growth, biofilm formation and structure were evaluated using microplate Alamar Blue assay, spectrophotometric procedure and scanning electron microscopy (SEM), respectively. Involvement of reactive oxygen species in CSC-mediated biofilm formation was investigated by including catalase in biofilm-forming cultures.

**Results:**

CSC did not affect the growth of planktonic bacteria, but rather led to a statistically significant increase in biofilm formation at concentrations of 0.4-3.12 mg/L, as well as in the viability of biofilm-forming bacteria at CSC concentrations of 0.2-1.56 mg/L. SEM confirmed an agglomerated biofilm matrix and irregular bacterial morphology in CSC-treated biofilms. Inclusion of catalase caused significant attenuation of CSC-mediated augmentation of biofilm formation by *Mtb*, implying involvement of oxidative stress. These findings demonstrate that exposure of *Mtb* to CSC resulted in increased biofilm formation that appeared to be mediated, at least in part, by oxidative stress, while no effect on planktonic cultures was observed.

**Conclusion:**

Smoking-related augmentation of biofilm formation by *Mtb* may contribute to persistence of the pathogen, predisposing to disease reactivation and counteracting the efficacy of antimicrobial chemotherapy.

## 1. Introduction

Cigarette smoke (CS) exposure has been identified as one of the major risk factors associated with the high morbidity and mortality associated with pulmonary tuberculosis (TB) [1–4]. CS exposure weakens the pulmonary immune system [5], compromising the protective activity of macrophages, resulting in decreased production of proinflammatory cytokines [4] and recruitment of T cells [6, 7]. In the case of *Mycobacterium tuberculosis* (*Mtb*), smoking-associated immune dysfunction promotes bacterial survival in macrophages [8, 9]. Additionally, CS exposure prevents granuloma formation [1, 3, 6], leading to accelerated disease severity and progression [5, 10]. The mechanisms of CS-mediated exacerbation of disease severity have been largely attributed to weakening of the immune system with little attention focused on the direct effects of CS exposure on the bacterial pathogen.

During infection, a mixture of heterogenous populations of *Mtb* organisms is found in TB lesions, with actively-replicating(AR) bacilli located predominantly in macrophages, while persistent, slow-replicating (SR) and dormant, non-replicating(NR) organisms are found in the central foci of granuloma lesions [11–14] and AR in macrophages that accumulate at the peripheral rim of the granuloma [13]. With respect to the *in vitro* setting, the AR, SR, and NR *Mtb* populations are found predominantly in planktonic, biofilm-forming and preformed biofilm cultures, respectively [15, 16].

The effects of CS exposure on planktonic and biofilm cultures of bacteria other than *Mtb* have been described. For example, in the case of *Bifidobacterium animalis*, cigarette smoke condensate (CSC) exposure was found to inhibit bacterial growth [17], while in other bacterial genera, including *Staphylococcus aureus*, *Pseudomonas aeruginosa*, and *Streptococcal* species, CSC exposure resulted in induction of biofilm formation, without affecting bacterial growth [18–20]. Increased biofilm formation by these CSC-exposed organisms has been attributed to stress induced by the high content of reactive oxygen species (ROS) [20–22] and other toxicants in CSC such as iron [23] and nicotine [20], which also lead to production of ROS [24].

The term ROS encompasses various potent oxidants, including superoxide (O_2_^−^), hydrogen peroxide (H_2_O_2_), and hydroxyl radical (HO^−^) that lead to oxidative stress when produced excessively [25]. Bacteria respond to oxidative stress by forming biofilm, which enables adaptation to harsh environments [26]. For example, *P*. *aeruginosa* and *Escherichia coli* respond to ROS by producing extrapolymeric substances (EPS), which, in the case of *E*. *coli*, results in the accumulation of these EPS at the air-liquid interphase rather than the interior of the biofilm [27]. Moreover, varying levels of ROS encountered in different sectors of the biofilm mass result in the establishment of heterogenous microenvironments, enabling bacterial cells to alter their metabolic rates accordingly, resulting in the formation micropopulations that consist predominantly of SR and NR organisms [26].

In the current study, the effects of exposure of *Mtb* to CSC on the growth and viability of AR and SR bacteria found in planktonic and biofilm-forming cultures, respectively, have been evaluated. This was achieved by assessing bacterial growth and viability using the microplate Alamar Blue assay method and a colony-counting procedure, respectively. Biofilm formation and structure were evaluated using a crystal violet-based spectrophotometric procedure and scanning electron microscopy (SEM), respectively. The possible involvement of ROS in CSC-mediated biofilm formation was determined by inclusion of catalase in the culture media prior to exposure of *Mtb* to CSC.

## 2. Materials and Methods

### 2.1. Bacterial Strain and Growth Media

The *Mtb* H37Rv strain (ATCC: 25618) known to be sensitive to all primary anti-TB drugs was used as the test strain for the investigations described below.

Beckton Dickinson (BD) Difco Middlebrook 7H10 agar (BD Difco, Diagnostics, Sparks, MD, USA) containing 0.5% glycerol, 10% oleic acid, dextrose, catalase (OADC) and BD Middlebrook 7H9 broth supplemented with 10% OADC, 0.2% glycerol (OG) with or without 0.05% Tween 80 (T), referred to hereafter as 7H9-OGT and 7H9-OG, respectively, were prepared according to the manufacturers' instructions. Sauton broth medium was prepared as described [28].

### 2.2. Chemicals and Reagents

Unless otherwise stated, most chemicals and reagents were purchased from the Sigma-Aldrich Chemical Co (St. Louis, MO, USA), LASEC (Johannesburg, South Africa), Whitehead Scientific (Johannesburg, South Africa), Beckton Dickinson (Johannesburg, South Africa), and Merck (Johannesburg, South Africa).

### 2.3. Cigarette Smoke Condensate (CSC) and Catalase

CSC was purchased from Murty Pharmaceuticals (Lexington, KY, USA) as a 40 g/L stock solution prepared in 100% dimethyl sulfoxide (DMSO). The working concentrations of CSC, prepared in double dilutions in DMSO ranged from 0.05 to 3.12 mg/L. For all CSC experiments, DMSO was added to the various control systems at a final concentration of 1%, which was the maximum used and had no adverse effects on the bacteria in any of the assays. One set of controls (DMSO free) was treated with phosphate-buffered saline (PBS, pH 7.4) instead of DMSO. Catalase from bovine liver was used at a fixed, final concentration of 100 mg/L in assays of biofilm formation.

### 2.4. Preparation of Inoculum

A bacterial inoculum was prepared as described, with minor modifications [16]. Briefly, a seed culture of *Mtb* cells was inoculated into 50 mL of 7H9-OGT broth and grown to mid-log phase at 37°C under stirring conditions. The bacterial cells were harvested by centrifugation at 2851 x *g* at room temperature (RT) for 15 minutes (min) and the supernatant discarded. The pellet was washed twice and resuspended in 7H9-OG, followed by adjustment of the optical density (OD) to 0.6 at 540 nm yielding ca. 10^7^-10^8^ colony-forming units (CFU)/mL. The bacterial inoculum was used at approximately 10^5^ CFU/mL in all of the assays described below.

### 2.5. Preparation of Cultures

Cultures were prepared by adding 7H9-OGT or Sauton broth media to the wells of 96- or 24-well microtissue culture plates at final volumes of 0.1 or 2 mL/well for planktonic and biofilm-forming bacterial cultures, respectively, followed by addition of the bacterial cells. The contents of the wells were thoroughly mixed and the plates incubated at 37°C in the dark for seven days with frequent mixing every two days for planktonic cultures, while the biofilm plates were wrapped in parafilm and incubated for five weeks without shaking in the presence of 5% CO_2_.

### 2.6. Determination of Bacterial Growth

For assays of planktonic growth, cultures were prepared as described above, followed by addition of various concentrations of CSC, and bacterial growth was determined by the Alamar Blue method [29]. The plates were incubated for six days and Alamar Blue solution (10%, final) was added to each well and the plates incubated for a further 24-hour (h) period to allow for a change in colour from blue to pink in growing cultures. The effect of CSC on bacterial growth was evaluated by monitoring change in colour of the Alamar Blue dye in the CSC-untreated and CSC-treated cultures as described [29].

For assays of biofilm formation, the cultures with and without added CSC, were prepared and incubated as above. Biofilm formation was detected visually by the development of a white layer with an irregular rough appearance on the surface of the growth medium and quantified at the end of week five.

#### 2.6.1. Biofilm Quantification

The amounts of biofilm biomass in the CSC-treated and control cultures were quantified using a crystal violet-based staining procedure as described [16] with minor modifications. The supernatants, containing planktonic cells in the biofilm-forming cultures, were removed, and the residual biomass in the wells was washed once with 1 mL distilled water and air dried. The residual matrix was stained with 1 mL of 1% crystal violet and incubated for 30 min at room temperature (RT) followed by three washes with 1 mL distilled water to remove the unbound crystal violet dye and air dried. The biofilm-associated crystal violet was then extracted with 1 mL of 70% ethanol, followed by 10-fold dilution and measurement of OD at 570 nm using a Spectronic Helios UV-Vis spectrophotometer (Merck, USA).

### 2.7. Determination of Bacterial Survival

Bacterial viability was determined using a colony-counting procedure as described [16, 30]. The cultures were prepared as for measurement of growth.

For planktonic cultures, the contents of each well were thoroughy mixed and sampled and serial 10-fold dilutions were prepared in PBS, followed by plating on 7H10 agar medium for the development of colonies.

In the case of the biofilm-forming cultures, prior to plating, the biofilm-encased cells were released into the growth medium by dissolving the biofilm matrix in each well with Tween 80 (0.05% final) under shaking conditions at 37°C for 6 h. The contents of the wells were then plated as described for planktonic cultures.

The control and CSC-exposed cultures were plated on the initial and last days of each experiment, and these time points were recorded as day zero (D0) and day seven (D7) and week zero (W0) and week five (W5) for planktonic and biofilm cultures, respectively. The colonies were counted and the numbers of bacteria (CFU/mL) were determined.

### 2.8. Measurement of Extracellular pH Levels

Measurement of the pH of the growth medium was undertaken as an additional, albeit indirect assessment, of the effects of the CSC on bacterial growth. The pH levels in the culture media were measured directly using the Jenway 3520 pH/Mv/Temperature Meter (LASEC, Johannesburg, South Africa) following the manufacturer's instructions at the initial and end time points of the experiments.

### 2.9. Catalase Activity

The effect of ROS on biofilm formation was determined using added catalase. Biofilm cultures were prepared as for bacterial growth in the absence and presence of a fixed concentration of catalase (100 mg/L) followed by treatment of cultures with various concentrations of CSC (0.78-3.12 mg/L). The protective potential of catalase was determined by comparing the extent of biofilm formation by the CSC-untreated and CSC-treated systems in the absence and presence of catalase.

### 2.10. Scanning Electron Microscopy (SEM)

SEM was performed on biofilm-forming cultures as described [31, 32] with minor modifications. The cultures were prepared as described, and the supernatants were removed from wells. The biofilm biomass residues were fixed with 1 mL of 2.5% glutaraldehyde/formaldehyde (GA/FA) fixative for 24 h, and the contents of the wells washed three times with PBS (pH 7.4) for 10 min. The biofilm biomass was progressively dehydrated in a graded series of increasing ethanol concentrations (30%, 50%, 70%, 90%, and 3x 100% ethanol) for 10 min each, followed by treatment with a mixture of hexamethyldisilazane (HMDS) : ethanol (1 : 1 *v*/*v*) and 100% HMDS for 1 h each and finally by the addition of 100% HMDS for overnight drying. The biofilm biomass was then transferred onto double-sided carbon tape (SPI Supplies) and mounted onto aluminium stubs and carbon coated using an EMITECH K950X instrument (Quorum Technologies). The biofilm structure micrographs were analysed using a Zeiss (Oberkochen, Germany) Ultra Plus field emission gun scanning electron microscope (FEG-SEM). The effect of CSC on biofilm morphology was evaluated by comparing the images of CSC-treated cultures with those of the CSC-untreated controls (W5).

### 2.11. Statistical Analysis

Statistical analyses were performed on all data using the GraphPad Instat 3 Programme, and the results expressed as the mean values ± standard deviations (SDs). Comparisons between CSC-nonexposed and CSC-exposed and catalase-untreated and catalase-treated cultures were performed using the unpaired *t*-test/Mann–Whitney *U*-test. For each assay, three sets of experiments with triplicate determinations for each solvent control and CSC-treated system with and without catalase were included.

## 3. Results

### 3.1. Effect of CSC on Bacterial Growth and Biofilm Formation

In the case of planktonic growth, no effects of CSC were observed (data not shown).

**Figure 1:**
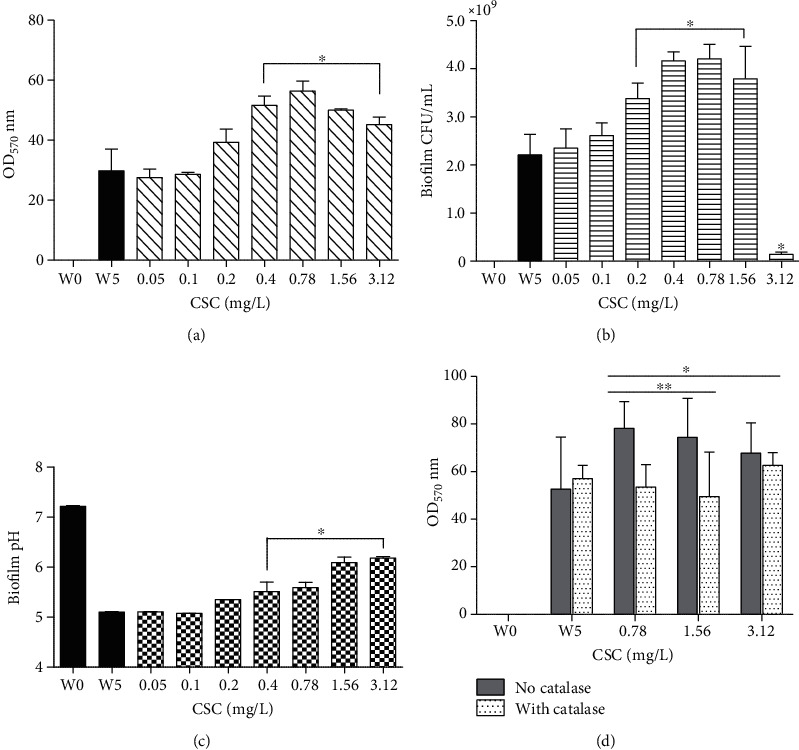
The effect of various concentrations of cigarette smoke condensate (CSC) on biofilm-forming cultures of *Mtb.* (a) Biofilm was measured using the crystal violet spectrophotometric procedure. (b) Viability of biofilm-forming *Mtb* was determined using a colony-counting procedure and the results are presented on a linear graph. (c) Measurement of the pH levels of the bacterial growth medium. (d) The effect of catalase (100 mg/L) on biofilm formation by control and CSC-treated *Mtb* using the crystal violet spectrophotometric procedure. The results of three separate experiments, each with triplicate determinations, are presented as the mean values ± SDs. (a–c) The black and striped/lined/checkered bars represent the CSC-untreated control (W5) and CSC-treated cultures, respectively, while for (d) the panels on the left (grey columns) and right of each pair (dotted columns) represent catalase-untreated, catalase-treated, CSC-treated cultures, respectively. Statistical significance is represented by an asterisk (^∗^*P* value *<* 0.05). For (d), ^∗^ represents concentrations of CSC which induced significant increases in biofilm formation in the absence of catalase, while ^∗∗^ represents significant inhibition of the CSC-mediated increases in biofilm formation in the presence of catalase for each CSC concentration.

However, as shown in Figure 1(a), exposure of biofilm-forming bacteria to CSC resulted in a statistically significant, dose-dependent increase in biofilm formation, which was evident at ≥0.2 mg/L of CSC (*P* ≤ 0.05) and maximal at 0.78 mg/L, declining slightly thereafter.

### 3.2. Effect of CSC on Bacterial Viability

For planktonic organisms, in the absence of CSC, the number of bacteria increased from 2.1 × 10^5^ ± 3.6 × 10^4^ CFU/mL at D0 to 1.93 × 10^8^ ± 6.7 × 10^8^ CFU/mL and remained unchanged in the presence of CSC at D7.

In the case of biofilm-forming cultures as shown in Figure 1(b), the number of bacteria in the CSC-free control system increased from 1.55 × 10^5^ ± 1.4 × 10^4^ CFU/mL at W0 to 1.38 × 10^9^ ± 1.5 × 10^9^ CFU/mL at W5, while exposure to CSC at concentrations of 0.2 mg/L and 1.5 mg/L, resulted in statistically significant augmentation of growth that declined significantly at concentrations of ≥3.12 mg/L.

### 3.3. Effect of CSC on pH of the Growth Media

In planktonic cultures, the pH decreased from 6.8 to 6.66 ± 0.006 and to 6.67 ± 0.005 during bacterial growth in the absence and presence of CSC, respectively (not significantly different).

In the case of biofilm-forming cultures as shown in Figure 1(c), the pH of the CSC-untreated control cultures decreased from 7.22 ± 0.017 at W0 to 5.27 ± 0.17 at W5, while in the presence of CSC, the decline in pH levels was partly attenuated, achieving statistical significance at concentrations of ≥0.4 mg/L CSC.

### 3.4. Catalase and Biofilm Formation

These results are shown in Figure 1(d). The inclusion of catalase in the biofilm-forming cultures resulted in significant attenuation of the CSC-mediated increase in biofilm formation, attaining statistical significance between the CSC-untreated and CSC-treated systems at CSC concentrations of 0.78-1.56 mg/L.

### 3.5. Scanning Electron Microscopy

**Figure 2:**
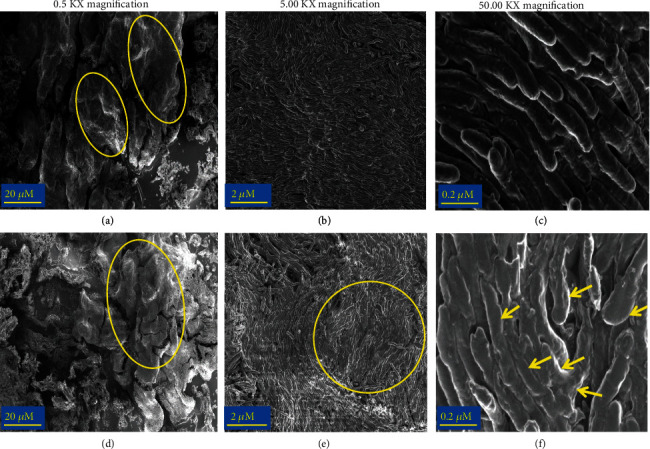
Bacterial morphology in the absence (a–c) and presence of CSC (d–f) using scanning electron microscopy (SEM). The results are a representative of three sets of experiments performed in duplicate. Panels on the left, middle, and right sides represent images taken at 0.5 KX, 5.00 KX, and 50.00 KX magnifications, for examination of biofilm integrity, cellular arrangements, and bacterial morphology, respectively. CSC-untreated control showing smooth intact regions of biofilm (a; oval areas), unidirectional cells (b), and elongated cells with interbacteria matrix material visible (c). CSC-treated cultures exposed to 0.7 mg/L CSC showing intact regions ((d) oval area), unidirectional cells coated with thick matrix ((e) yellow circle), and abnormally shaped cells surrounded by thicker more agglomerated matrix material ((f) arrows). The images were taken at 1 kV accelerating voltage, WD = 2.8 mm, with an InLens SE detector.

The effect of CSC on *Mtb* biofilm morphology was evaluated at CSC concentrations that augmented biofilm formation (0.2-0.7 mg/L) using SEM. In the absence of CSC, shown in Figures 2(a)–2(c), *Mtb* biofilm revealed a well-organised, intact structure, consisting of adjoined elongated rod-shaped cells, tightly bound side-by-side with extracellular matrix (ECM), arranged unidirectionally.

In the case of the cultures treated with CSC at 0.7 mg/L as shown in Figures 2(d)–2(f), gross biofilm morphology had a similar appearance to that of the CSC-untreated control systems, consisting of adjoined unidirectional cells. However, the ECM appeared more agglomerated than those of the controls, resulting in bacteria with an irregular morphology, covered by ECM. This distinction between CSC-untreated and CSC-treated systems was less pronounced at lower concentrations (0.2 and 0.4 mg/L) of CSC (data not shown).

## 4. Discussion

While the harmful effects of smoking on the immune system are well documented, there is limited information on how *Mtb* is affected by direct exposure to CS. In the current study, the effects of CSC on AR and SR *Mtb* organisms were evaluated *in vitro* using planktonic and biofilm-forming cultures, respectively.

In planktonic systems, bacteria grow in aerated, nutrient-rich environments that support the growth of AR organisms. In this setting, exposure to CSC had no significant effect on the growth and viability of planktonic bacteria or on the pH of the growth medium. Similar studies focused on other bacterial respiratory pathogens, such as *S*. *pneumoniae* and *S*. *aureus*, also reported that exposure of these organisms to CSC did not affect bacterial viability at concentrations of <200 mg/L [18, 19, 22]. The absence of inhibitory effects of CSC on the growth of *Mtb* in planktonic culture may relate to a more rapid growth rate and high-level production of secreted antioxidative enzymes such as superoxide dismutase, catalase, and peroxidases, as well as the presence of catalase and low molecular weight ROS scavengers, in the enriched Middlebrook 7H9 bacterial growth medium [25].

In the case of biofilm formation, in which *Mtb* was cultured in Sauton medium, exposure of the pathogen to CSC resulted in significant increases in biofilm formation, extracellular pH, and the number of viable bacteria. Elevated extracellular pH has been described previously to favor biofilm formation by bacterial pathogens other than *Mtb* [33], and is also conducive to bacterial replication [14]. These observations on CSC-mediated induction of biofilm formation are in agreement with studies reported by others for pathogens such as *S*. *pneumoniae* [18, 19] and *S*. *aureus* [22], in which exposure to CSC at concentrations of <200 mg/L resulted in increased production of biofilm. In the case of *S*. *aureus*, CSC-mediated biofilm formation was associated with increased numbers of bacteria in the biofilm fractions relative to those in the planktonic fractions of the cultures [22].

It is noteworthy that CSC-mediated augmentation of biofilm formation by *Mtb* was detected at concentrations of CSC, which are considerably lower than those that activate biofilm formation by other respiratory pathogens such as the pneumococcus [18, 19]. Although unexplained, this may be due to a slower induction of protective antioxidative responses following exposure of slow-growing *Mtb* to stressors such as CSC.

Ultrastructural analysis using SEM revealed that CSC-mediated enhancement of biofilm formation by *Mtb* did not alter biofilm structural integrity, maintaining unidirectional adjoined bacterial arrangements. Exposure of the pathogen to CSC did, however, result in biofilm-associated irregular morphological changes of the bacteria, characterised by abnormally shaped cells coated with thicker matrices. This could potentially lead to increased bacterial survival and tolerance to external factors, including host anti-infective defense mechanisms, as well as antibiotics [34].

To probe the possible involvement of CSC-associated ROS on biofilm formation by *Mtb*, the effects of inclusion of catalase were investigated. In this context, it is noteworthy that H_2_O_2_ has been shown to increase biofilm formation by many types of bacteria including *P*. *aeruginosa* and *S*. *aureus* [20–22]. In the current study, addition of catalase, a known antioxidant enzyme, which hydrolyses the stable, cell-penetrating ROS, H_2_O_2_, during exposure of *Mtb* to CSC, resulted in the attenuation of CSC-mediated biofilm formation. While clearly implicating CSC-derived H_2_O_2_ as a major stressor triggering biofilm formation by *Mtb*, we do concede that toxicants present in CSC, other than H_2_O_2_, may also induce biofilm formation by the bacterial pathogen as reported for other organisms [20, 23, 24].

In conclusion, the current study has demonstrated effects of CSC on biofilm formation by *Mtb* that may enable the pathogen to evade host defense mechanisms, leading to bacterial survival and persistence, favoring bacterial replication, which may enable reactivation of disease and bacterial tolerance to antibiotics through increased biofilm formation.

## Data Availability

All data generated and analysed in this study have been included in this publication and will be available from the corresponding author upon request.
